# Polymorphic nanobody crystals as long‐acting intravitreal therapy for wet age‐related macular degeneration

**DOI:** 10.1002/btm2.10523

**Published:** 2023-05-04

**Authors:** Shuqian Zhu, Shilong Fan, Tianxin Tang, Jinliang Huang, Heng Zhou, Chengnan Huang, Youxin Chen, Feng Qian

**Affiliations:** ^1^ School of Pharmaceutical Sciences, Beijing Advanced Innovation Center for Structural Biology, and Key Laboratory of Bioorganic Phosphorus Chemistry and Chemical Biology (Ministry of Education) Tsinghua University Beijing People's Republic of China; ^2^ Beijing Frontier Research Center for Biological Structure Tsinghua University Beijing People's Republic of China; ^3^ Quaerite Biopharm Research Beijing People's Republic of China; ^4^ Shuimu BioSciences Co. Ltd. Beijing People's Republic of China; ^5^ Peking Union Medical College Hospital Beijing People's Republic of China

**Keywords:** crystal suspension, nanobody, thermodynamic properties, wet AMD

## Abstract

Wet age‐related macular degeneration (wet AMD) is the most common cause of blindness, and chronic intravitreal injection of anti‐vascular endothelial growth factor (VEGF) proteins has been the dominant therapeutic approach. Less intravitreal injection and a prolonged inter‐injection interval are the main drivers behind new wet AMD drug innovations. By rationally engineering the surface residues of a model anti‐VEGF nanobody, we obtained a series of anti‐VEGF nanobodies with identical protein structures and VEGF binding affinities, while drastically different crystallization propensities and crystal lattice structures. Among these nanobody crystals, the *P*2_1_2_1_2_1_ lattice appeared to be denser and released protein slower than the *P*1 lattice, while nanobody crystals embedding zinc coordination further slowed the protein release rate. The polymorphic protein crystals could be a potentially breakthrough strategy for chronic intravitreal administration of anti‐VEGF proteins.

## 1 INTRODUCTION

Age‐related macular degeneration (AMD) is a degenerative disease that affects the central retina in patients above 50 years of age, and the disease risks steeply increase to 20% after 70.^1^ Accounting for 8.7% of blindness cases, AMD is considered the most common cause of visual impairment. By 2040, the projected number of AMD patients will approach ~288 million, a heavy health care burden worldwide.^2^ Wet AMD, a malignant and advanced subtype of clinical AMD, causes 90% drastic visual impairment in AMD patients because of its rapid disease progression.^3^

As the development of wet AMD progresses, the new vessels gradually invade Bruch's membrane and the retinal pigment epithelium (RPE) layer and stimulate excessive secretion of vascular endothelial growth factor (VEGF) by RPE cells, which results in aberrant proliferation of the choriocapillaris, called choroidal neovascularization. These new immature vessels may cause plasma leakage or hemorrhages, creating detachment of the neuroretina or RPE from Bruch's membrane, leading to severe central vision loss in weeks or months.^1^, ^4^, ^5^, ^6^, ^7^ Anti‐VEGF proteins have been the cornerstone of wet AMD therapeutics, and approved anti‐VEGF proteins include ranibizumab, aflibercept, conbercept, brolucizumab, and bevacizumab (off‐label use).^8^, ^9^, ^10^

Regardless of the molecular design, a central theme of anti‐VEGF protein therapeutics is to achieve a prolonged injection interval and avoid frequent intravitreal injection.^11^ The near‐future goal in industry is to lengthen the injection interval from the current ~2 months (aflibercept, 2 mg dose) to 3–4 months (aflibercept, 8 mg dose phase III clinical trial) by drastically increasing the injection dose.^12^, ^13^ However, with a strict limitation of injection volume (<100 μl) and needle size (30 G or smaller), the injectable protein dose in solution quickly reaches its ceiling due to inadequate protein stability and exponentially increased viscosity when the protein concentration is >100 mg/ml due to the intrinsically large, complex and flexible protein structure.^14^ Furthermore, considering the first‐order drug elimination pharmacokinetics from vitreous humor into aqueous humor,^15^, ^16^, ^17^ simply increasing the injection dose is not a highly effective strategy for prolonging the injection interval. Theoretically, the injection interval will only increase one elimination half‐life (8–11 days) if the injection dose is doubled.

We engineered a series of crystallizable anti‐VEGF proteins, which formed pseudo “polymorphic” crystal lattice structures. Some of the crystalline anti‐VEGF proteins could act as potential long‐acting intravitreal protein therapeutics for wet AMD, based on the following intrinsic characteristics: (1) As a thermodynamically more stable system, protein crystals are naturally more stable than protein solution, which has been proven experimentally.^18^ (2) High concentration protein crystal suspension is expected to have lower viscosity and thus better injectability than a protein solution with the same concentration.^19^, ^20^ (3) More importantly, protein release will be hindered by the crystal structure; therefore, the overall intravitreal protein retention will be modulated by the crystal lattice as well as the first‐order elimination, which could significantly increase the injection interval.

To modulate the protein crystal lattice energy through surface mutation, one of the strategies we employed was the “surface entropy reduction (SER)” hypothesis. Derewenda et al. proposed that the microscopic state of the protein surface plays a critical role in protein crystallization.^21^, ^22^, ^23^ The solvent‐exposed residues with large hydrophilic side chains exposed to solvents, such as Lys, Glu, and Gln, possess high conformational entropy, which is more likely to hamper the crystallization of proteins by enhancing the surface entropy shield during protein molecule packing. Replacing amino acids with Ala, which has lower conformational entropy, can significantly promote protein crystallinity. This approach, called SER, has been well verified on many proteins.^24^, ^25^, ^26^ Therefore, the SER hypothesis provided an available perspective to induce different crystal lattice arrangements for fluctuations in crystal lattice entropy. On the other hand, the choice of crystal lattice is inextricably linked with the interaction among the contacting interface residues. Modification of the interaction by rational mutation at the contact interface would drive the protein molecules to occupy the new position in the crystal lattice, leading to different protein matrices.

The significantly lower viscosity, superior stability, and potential long‐acting release property make crystalline protein a potential breakthrough for novel wet AMD therapy. Here, we successfully changed the crystal lattice arrangement of therapeutic nanobody (termed mNb‐WT) by rational mutations of surface residues, which remarkably reduced the dissolution rate of protein molecules from the crystal into the solvent. By modulating the solvent compositions, the dissolution rate could be delayed further. We believe that these findings will provide a new strategy for intravitreal sustained administration in wet AMD treatment.

## 2 RESULTS

### 2.1 Theoretical evaluation

The clearance of protein solution in the eye, as the comparatively isolated environment, corresponded to the primary clearance kinetic equation (1).

(1) $${\text{Rate}}_{\text{elimination}}={\mathrm{kC}}_{t}$$

where $C_{t}$ is the transient concentration of the protein drug in the vitreous humor of the eye and k is the primary clearance constant. When crystalline protein is introduced, the clearance process of the protein drug from the eye could be considered as two‐compartment model, and it undergoes three steps: (1) the release of protein molecules from the crystal surface; (2) the diffusion of protein molecules from the crystal surface to the entire vitreous humor of the eye and reach equilibrium; and (3) the first‐order clearance of protein molecules from the vitreous humor into the aqueous humor. Step 2 was determined by the Noyes–Whitney dissolution equation (2)^27^:

(2) $${\text{Rate}}_{\text{dissolution}}=A∙k_{D}∙\left(C_{s}-C_{t}\right)/\mathrm{VL}$$

where $C_{s}$ is the equilibrium solubility of protein molecules in certain crystal lattice, $k_{D}$ is the diffusion rate constant of protein molecules in the vitreous humor, A is the surface area of the crystal, V is the volume of the intravitreal aqueous volume, and L is the thickness of the diffusion layer. Thus, the net intravitreal protein concentration (*C*) generated by the crystal suspension in the eye can be qualitatively rewritten as follows:

(3) $$\text{Rate}={\text{Rate}}_{\text{dissolution}}-{\text{Rate}}_{\text{elimination}}=A∙k_{D}∙\left(C_{s}-C_{t}\right)/\mathrm{VL}-kC_{t}$$

Therefore, the differences in the protein crystal lattice of the same protein molecules could profoundly disturb the molecular clearance of the protein drug in the vitreous humor, through the modulation of the equilibrium solubility of crystalline of protein.

### 2.2 Lattice arrangement of protein crystals formed by mNb‐WT molecules

**FIGURE 1:**
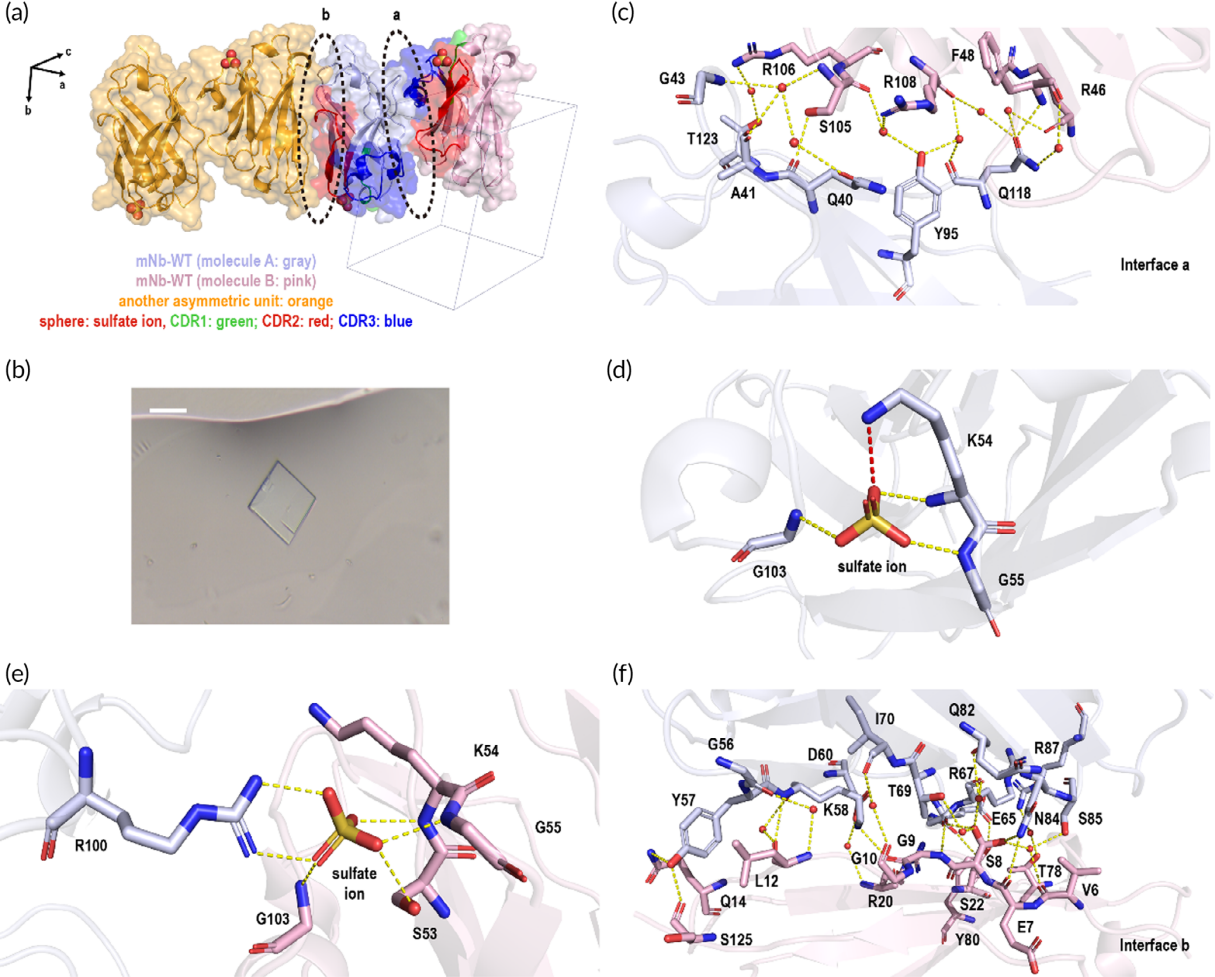
The crystal lattice and morphology of protein crystals formed by model nanobody mNb‐WT. (a) The surface and cartoon model of protein arrangement in the crystal lattice. The lattice axes are shown as black arrows. The two molecules in the asymmetric unit are gray and pink, and the other asymmetric unit is shown in orange. The CDR region is shown in green, red, and blue, and the sphere model shows the sulfate ions in the structure. (b) The crystal morphology of mNb‐WT. The scale bar represents 20 μm. (c) The interaction between two protein molecules inside the asymmetric unit. (d) and (e) The sulfate ion binding sites of mNb‐WT. The salt bridge is colored red, and the hydrogen bonds are yellow. (f) The interaction between two asymmetric units.

We dissected the molecular structure and crystal lattice arrangement of mNb‐WT by single crystal X‐ray diffraction. mNb‐WT protein molecules were arranged in the *P*1 lattice inside the lamellar crystals with two protein molecules as one asymmetric unit (Figure 1a,b). Up to a 1.27 Å resolution (Table S1), we could clearly see the participation of water and sulfate ions during crystal packing in the solvent. Water molecules mediate a strong hydrogen bond network to stabilize two protein molecules inside the asymmetric unit (interface a) (Figure 1c). Meanwhile, the sulfate ions also contributed to the protein stability in the crystal lattice, both by affecting the protein itself and by enhancing interactions between two protein molecules that belong to different asymmetric units (Figure 1d,e). In addition to sulfate ions, water still played an essential role in contributing to protein packing in the crystal lattice to various degrees at the contact interfaces among the asymmetric units, such as interface b (Figure 1f).

Because of the small size and compact structure, mNb‐WT proteins were tightly arranged in the *P*1 lattice, and the Matthews coefficient was 1.76 Å^3^/Da, corresponding to ~30% solvent content (Table 1).

**TABLE 1:** Crystal lattice comparison of mNb‐WT and mutations/analogues

Protein	mNb‐WT	Mutant 2	Mutant 7	Analogue 1	Analogue 2
PDB code	8IIU	8IJZ	8IJS	/	/
Space group	*P*1	*P*2_1_2_1_2_1_	*P*2_1_2_1_2_1_	*P*1	*P*1
Cell parameters	31.468	31.220	24.985	30.326	30.381
	39.369	41.090	53.267	38.419	38.201
	39.952	74.050	65.364	38.781	38.773
	102.140	90	90	101.846	102.137
	90.231	90	90	90.036	90.1
	90.948	90	90	92.225	92.211
Resolution (Å)	1.27	2.1	1.75	1.41	1.19
Cell volume (Å^3^)	48,380.15	94,993.55	86,662.6	44,186.2	43,958.52
Matthews coefficient (Å^3^/Da)	1.76	1.73	1.57	1.59	1.58
Number of copies	2	1	1	2	2
Solvent (%)	30	29	21.92	22.79	22.30

### 2.3 Rational design of nanobody mutants with intact VEGF binding affinities

**FIGURE 2:**
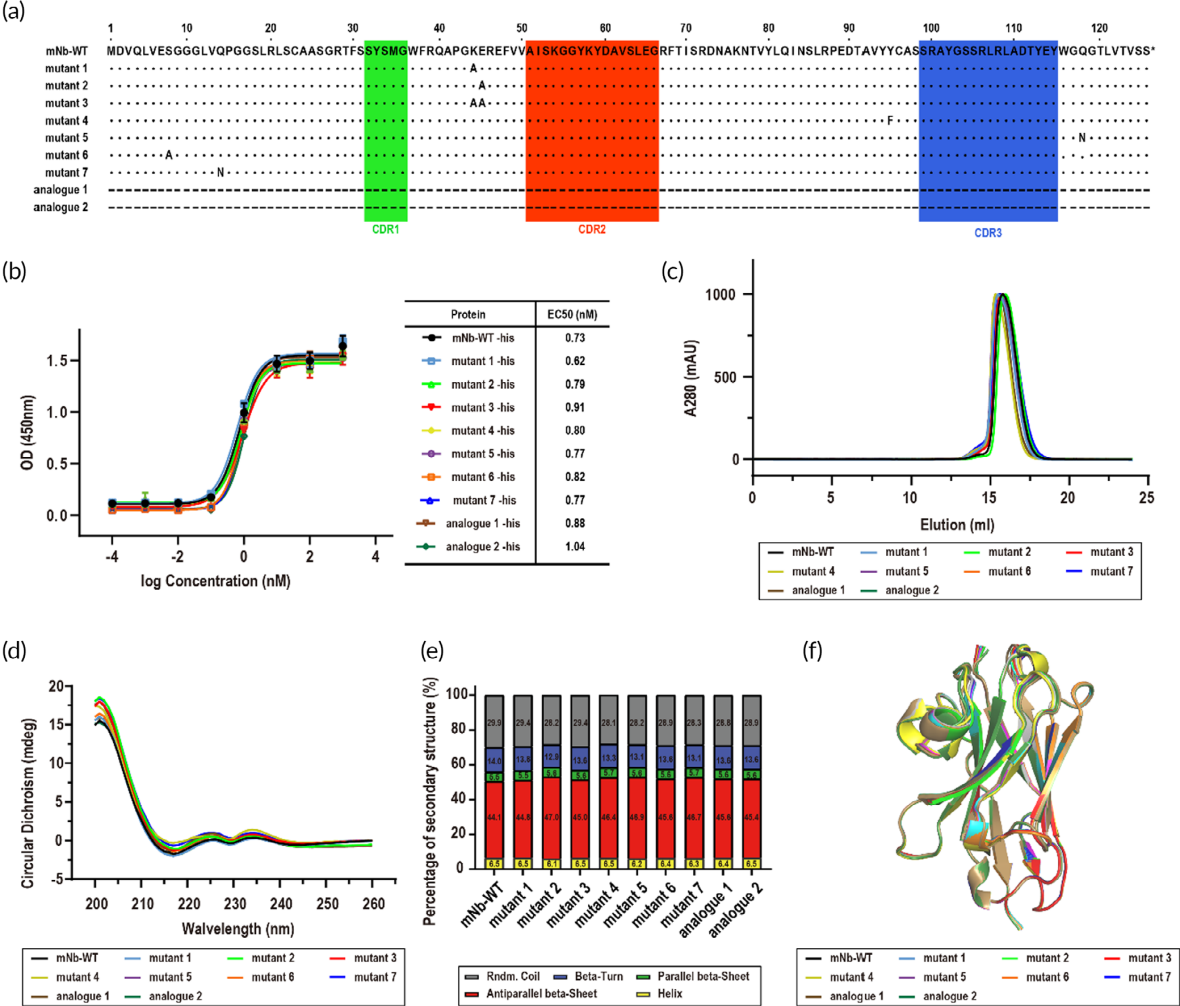
The sequence and pharmacological properties of rationally engineered mutants (mutants 1–7)/analogues (analogues 1–2) of mNb‐WT. (a) Rational mutation design based on the mNb‐WT crystal structure. (b) The vascular endothelial growth factor (VEGF) binding ability of mNb‐WT and mutants/analogues. (c) The normalized SEC curves of mNb‐WT and mutants/analogues. (d) The circular dichroism (CD) curves of mNb‐WT and mutants/analogues. (e) The percentage of the secondary structure of mNb‐WT and mutants/analogues in (d). No significance among the tested groups. (f) The alignment model of mNb‐WT and rational mutations predicted by AlphaFold.

To reduce the surface entropy of the entire protein according to the SER strategy, we analyzed the amino acids at the protein surface using the SER calculator web server and then determined the mutation candidates (K44 and E45) to improve the quality of the mNb‐WT crystal.^28^ Mutant 1 (K44A mutation), mutant 2 (E45A mutation), and mutant 3 (K44A/E45A mutation) were designed for the following experiments (Figure 2a). To weaken the interactions at the contact interface of the mNb‐WT nanobody in the *P*1 crystal lattice, residues at interface a and interface b (Figure 1a), which were representative of all the contact interfaces, were mutated to induce different crystal lattice arrangements. Mutant 4 (Y95F mutation), mutant 5 (Q118N mutation), mutant 6 (S8A mutation), and mutant 7 (Q14N mutation) were designed for crystal lattice comparison. With similar bioactivity to the mNb‐WT nanobody, two humanized anti‐VEGF nanobodies with mutation series, analogues 1 and 2, were tested together (Figure 2a). To remove the influence of protein posttranslational modifications, such as glycosylation and phosphorylation, all proteins were expressed in *Escherichia coli* without any purifying tag so that any of the discrepancies were induced by surface mutations of the mNb‐WT nanobody.

No significant difference was shown in the VEGF binding ability of mutants and analogues compared with mNb‐WT by ELISA (Figure 2b). The differences in EC_50_ were less than two‐fold, indicating that mNb‐WT and mutants/analogues showed equally potent binding capability to the VEGF‐A_165_ protein. The SEC comparison demonstrated that almost all the nanobodies were monomers in solvent; thus, the potential difference of the molecule packing in the crystal lattice reflects the performance of individual protein molecules rather than protein oligomers (Figure 2c). The secondary structure form detected by CD spectrum also showed no significant difference among mNb‐WT and mutants/analogues (Figure 2d,e). The overall structures predicted by the AlphaFold program for all of the nanobodies were highly similar to each other (Figure 2f). These results suggested that the nanobodies are all pharmaceutic analogues and can be considered the same protein molecules in drug administration.

### 2.4 Pseudo “polymorphic” crystal lattice formed by mNb‐WT and its mutants/analogues

**FIGURE 3:**
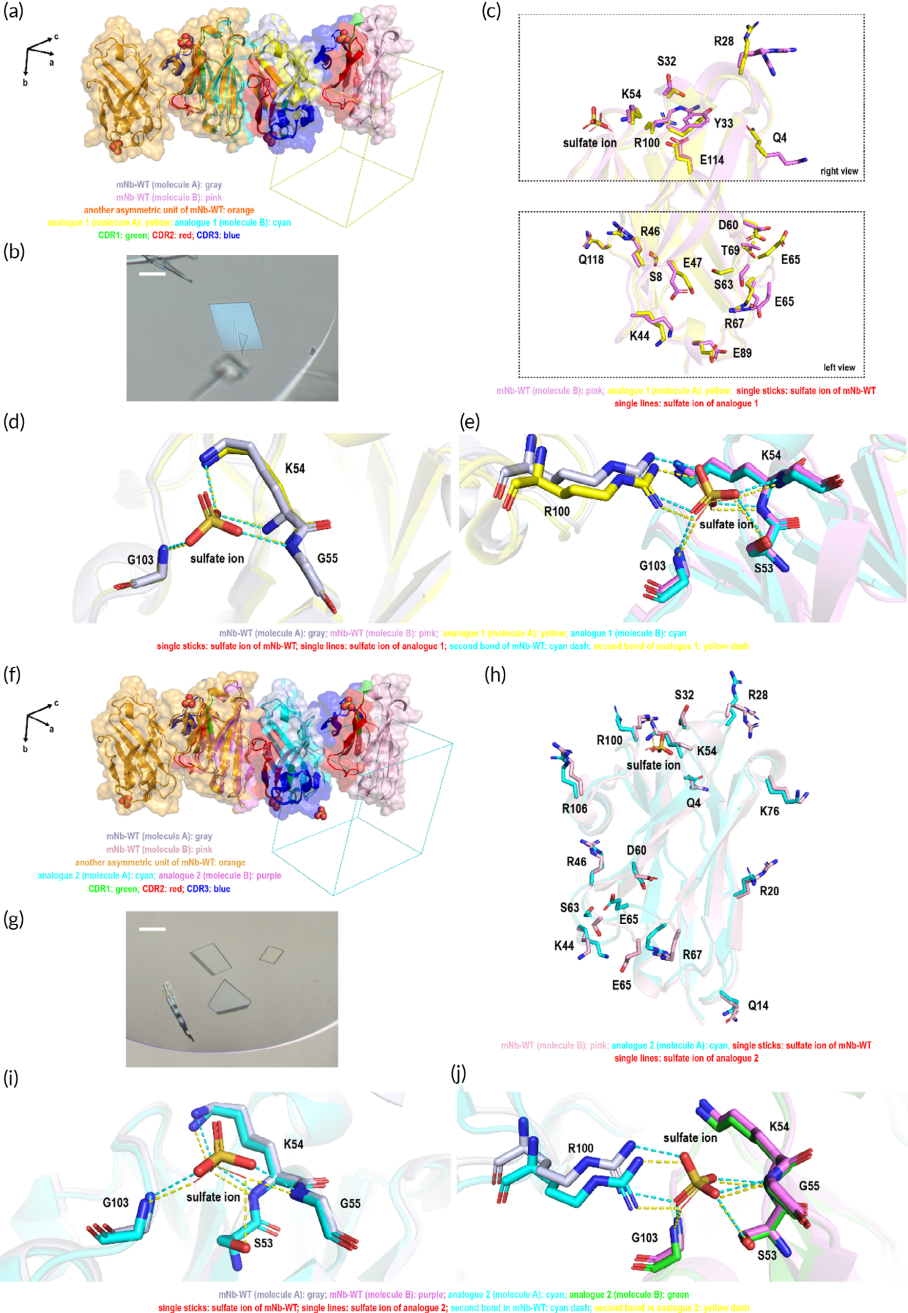
The crystal lattice and morphology of analogues 1 and 2. (a) Comparison of the protein arrangement in the crystal lattice of mNb‐WT and analogue 1. The lattice axes are shown as black arrows. (b) The crystal morphology of analogue 1. The scale bar represents 20 μm. (c) The conformational change of residues at the surface of mNb‐WT and analogue 1. The two protein molecules A and B of asymmetric units of mNb‐WT are shown in gray and pink, respectively; analogue 1 molecules are shown in yellow and cyan; CDR1, CDR2, and CDR3 are shown in green, red, and blue, respectively. (d, e) Comparison of the sulfate ion binding sites of mNb‐WT and analogue 1. (f) The protein arrangement in the crystal lattice of mNb‐WT and analogue 2. The lattice axes are shown as black arrows. (g) The crystal morphology of analogue 2. The scale bar represents 20 μm. (h) The conformational change of surface residues of mNb‐WT and analogue 2. The two protein molecules A and B of asymmetric units of mNb‐WT are shown in gray and pink, respectively; analogue 2 molecules are shown in cyan and purple; CDR1, CDR2, and CDR3 are shown in green, red, and blue, respectively. (i, j) Comparison of the sulfate ion binding sites of mNb‐WT and analogue 2.

With more mutations than mutants 1–7, analogues 1 and 2 showed similar crystal lattice and crystal morphology as mNb‐WT (Figure 3a,b and Figure 3f,g). Apparently, more conformational changes have emerged compared with mNb‐WT molecules in the crystal lattice as the results of the sequence differences (Figure 3c,h). The distinct wiggling of the whole side chains of several surface amino acids, even the turn of the terminal group of some side chains, contributed to the variation in the molecular interaction within the crystal lattice of analogues 1 and 2. The sulfate ion participation was similar to that of mNb‐WT (Figure 3d,e and Figure 3i,j), while the arrangements of sulfate ion sites in analogues 1 and 2 were more compact than mNb‐WT, indicating stronger interactions among side chains at sulfate ion sites and a higher density of crystals after mutation. This could also be confirmed by crystal lattice parameters, in which the Matthews coefficient of analogues 1 and 2 changed to 1.59 and 1.58 Å^3^/Da, respectively, and the solvent content was further reduced to 22.79 and 22.30% (Table 1).

**FIGURE 4:**
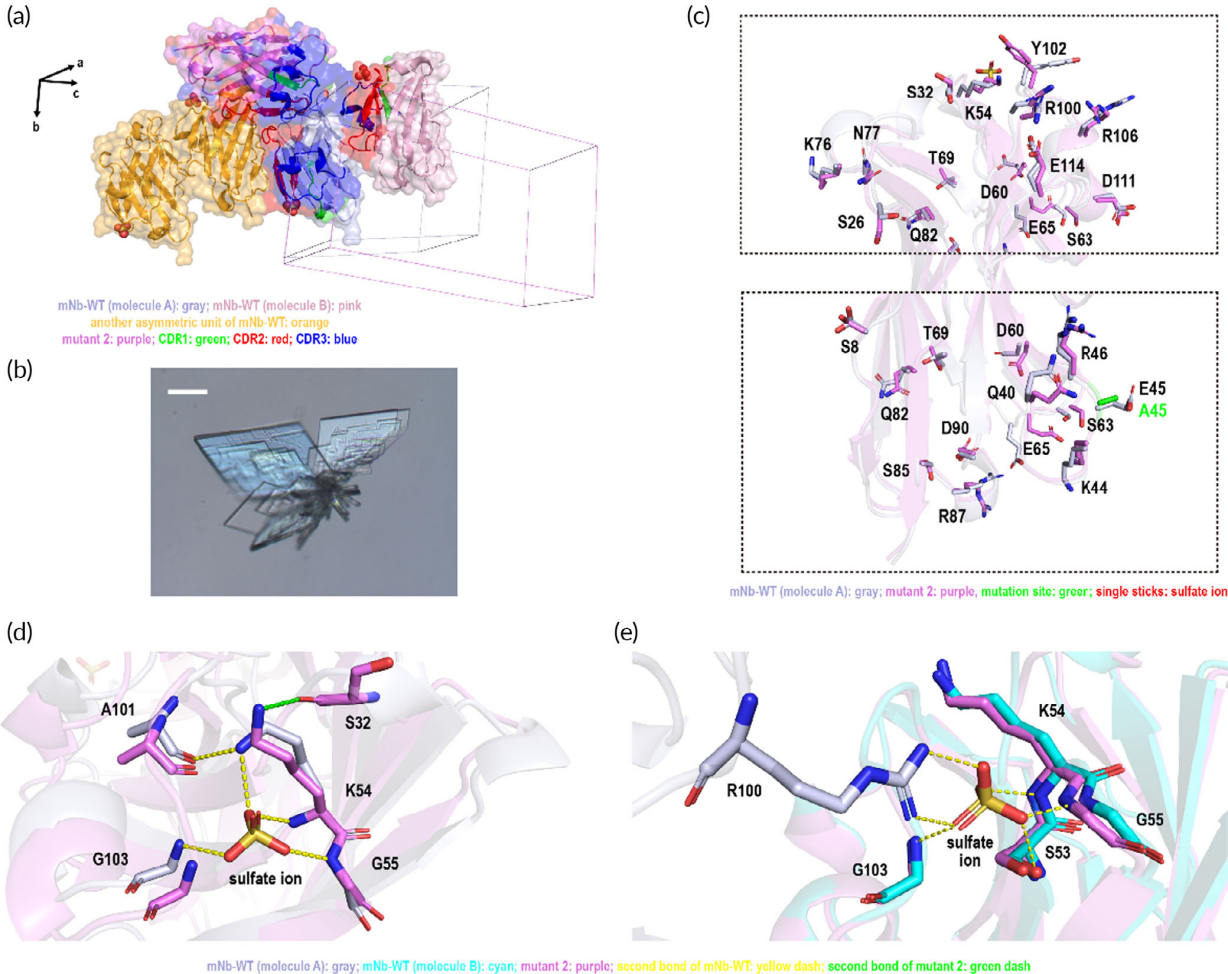
The crystal lattice alignment of mNb‐WT and mutant 2. (a) The protein arrangement in the crystal lattice of mNb‐WT and mutant 2. The crystal axes are shown as black arrows. (b) The crystal morphology of the mutant 2 crystal. (c) The conformational change of residues at the surface of mNb‐WT and mutant 2. The two protein molecules A and B of the asymmetric unit of mNb‐WT are shown in gray and pink, respectively; mutant 2 is shown in purple; CDR1, CDR2, and CDR3 are shown in green, red, and blue, respectively; and the mutation site is shown in green. (d, e) The conformational change of the surface residues at sulfate ion binding sites.

We dissected the crystal lattice arrangement of mutant 2 using the micro‐electron diffraction (microED) technique after treating cluster crystals by cryo‐FIB milling. We were surprised that mutant 2 was arranged in the *P*2_1_2_1_2_1_ lattice instead of the *P*1 group, with only one protein molecule as the asymmetric unit, although the crystal morphology was still lamellae, similar to mNb‐WT (Figure 4a,b). Many surface residues underwent dramatic conformational changes induced by only one residue mutation (Figure 4c). Unfortunately, we could not capture the electronic density map of water, thus unable to determine the participation of sulfate ions in the crystal lattice of mutant 2. However, if we overlaid mutant 2 molecules with molecule A of the mNb‐WT asymmetric unit, the terminal amino group of the K54 side chain showed an orientation dramatically opposed to that of mNb‐WT to form a hydrogen bond with the S32 residue (Figure 4d); however, all conformations of the S53, K54, G55, and G103 residues, which bond with the sulfate ion of molecule B in the mNb‐WT asymmetric unit, matched that of molecular conformation in the mutant 2 crystal (Figure 4e). These findings implied that the latter case was more likely if sulfate ions were involved in mutant 2 protein packing. Regardless of the sulfate ions' participation in protein lattice formation, the solvent content of 30% to 29% reduction suggested that the protein molecule of mutant 2 was denser than that of mNb‐WT (Table 1).

### 2.5 Zinc coordination enabled a densely packed pseudo “polymorphic” crystal

**FIGURE 5:**
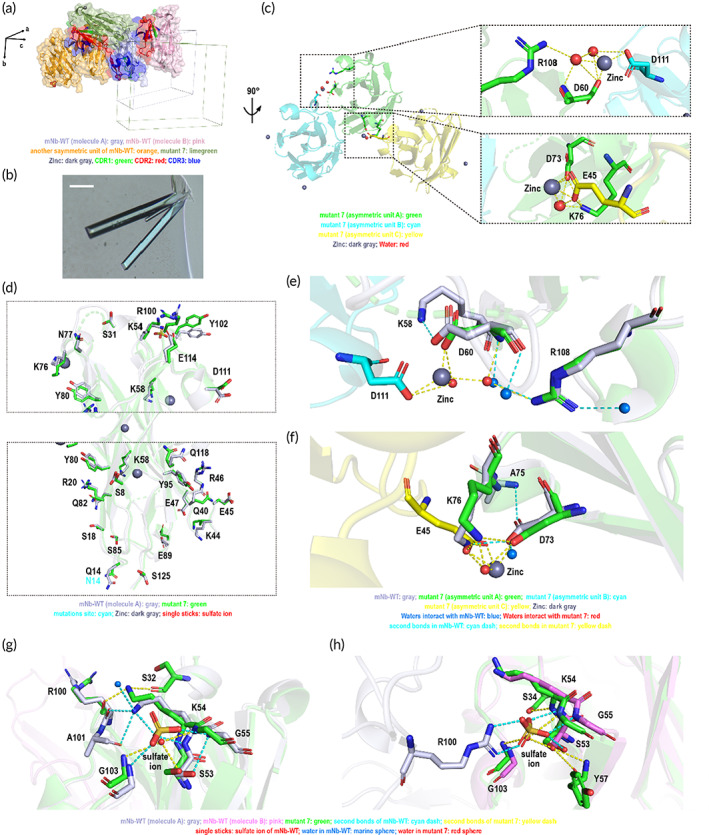
The crystal lattice arrangement of mNb‐WT and mutant 7. (a) The protein arrangement in the crystal lattice of mNb‐WT and mutant 7. The lattice axes are shown as black arrows. The two protein molecules A and B of mNb‐WT are shown in gray and pink, respectively; mutant 7 is shown in lime‐green, and CDR1, CDR2, and CDR3 are shown in green, red, and blue, respectively. (b) The crystal morphology of mutant 7. The scale bar represents 20 μm. (c) The zinc coordination units in the mutant 7 lattice. The zinc‐binding residues are shown in green, yellow and cyan for different molecules, yellow dashed lines indicate dipolar bonds and hydrogen bonds at the zinc‐binding unit, and water is shown as a red sphere. (d) The conformational change of the whole surface residues of mNb‐WT and mutant 7 molecules. The mutation sites are colored cyan. The details of residues at zinc binding sites of mNb‐WT and mutant 7 are shown in (e) and (f). The residues of mNb‐WT are shown in gray, and the green, cyan and orange residues belonged to mutant 7, of which orange residues were key residues with zinc binding in mutant 7 crystal. The red spheres are the waters in the mutant 7 lattice, and the blue spheres are in the mNb‐WT lattice. (g, h) Comparison of mNb‐WT and mutant 7 of the sulfate ion binding site.

Unexpectedly, mutant 7, replacing Q14 with N14, resulted in a radical change to the protein arrangement in the crystal lattice. The space group of mutant 7 turned into a *P*2_1_2_1_2_1_ lattice from the *P*1 lattice in mNb‐WT with only one protein molecule in the asymmetric unit (Figure 5a). The crystal morphology of mutant 7 was prismatic crystals instead of lamellae (Figure 5b). Unlike mNb‐WT crystals, mutant 7 recruited zinc ions rather than sulfate ions to help align protein molecules in the crystal lattice. Every mutant 7 molecule interacts with another asymmetric unit mediated by two zinc ions. At the first zinc binding site, the side chain of the D60 residue in asymmetric unit A, the side chain of the D111 residue in asymmetric unit B, and two water molecules formed a strong coordination unit with the zinc ion; meanwhile, the two water molecules also created typical hydrogen bonds with the D60 and R108 residues of asymmetric unit A and the D111 residue of asymmetric unit B to strengthen the periphery of the coordination unit (Figure 5c, top diagram). The other zinc coordination unit consisted of the side chains of residues D73 and K76 in asymmetric unit A, the side chain of residue E45 in asymmetric unit C, and one water molecule. Similar to the previous coordination unit, the hydrogen bonds formed among water molecule and the three key residues enhanced the stability of the coordination unit (Figure 5c, bottom diagram). Interestingly, these two coordination units were identical not to the conventional zinc binding motif, the classical zinc finger motif^29^ or histidine‐mediated zinc coordination among protein molecules,^30^ but rather to the central catalytic core of metal‐binding proteins, such as retroviral integrases.^31^

With just one carbon atom shorter than the Q14 side chain, the conformation of many surface residues of the mutant 7 (i.e., Q14N) protein was completely changed in the crystal lattice (Figure 5d). Through alignment with mNb‐WT, the interaction among residues in mNb‐WT at the same position as the first zinc binding site was found to be much looser, whereby D60 chose to interact with K58 in a more sprawling form and the R108 residue was forced to interact with water molecules to stabilize the side chain (Figure 5e). At the second zinc binding site position, the situation was very similar (Figure 5f). However, at the same position of the sulfate ion binding site of mNb‐WT, this did not occur. The sulfate ions were replaced by water molecules to connect these residues together in the same manner (Figure 5g,h). Although the interaction may be weaker than before, it indeed stabilized the residues to a similar extent.

Unexpectedly, the solvent content of the mutant 7 crystal was 21.92%, the minimum among the protein crystals, indicating the most compact molecular arrangement (Table 1).

### 2.6 Protein release kinetics from crystals were controlled by the crystal lattice

**FIGURE 6:**
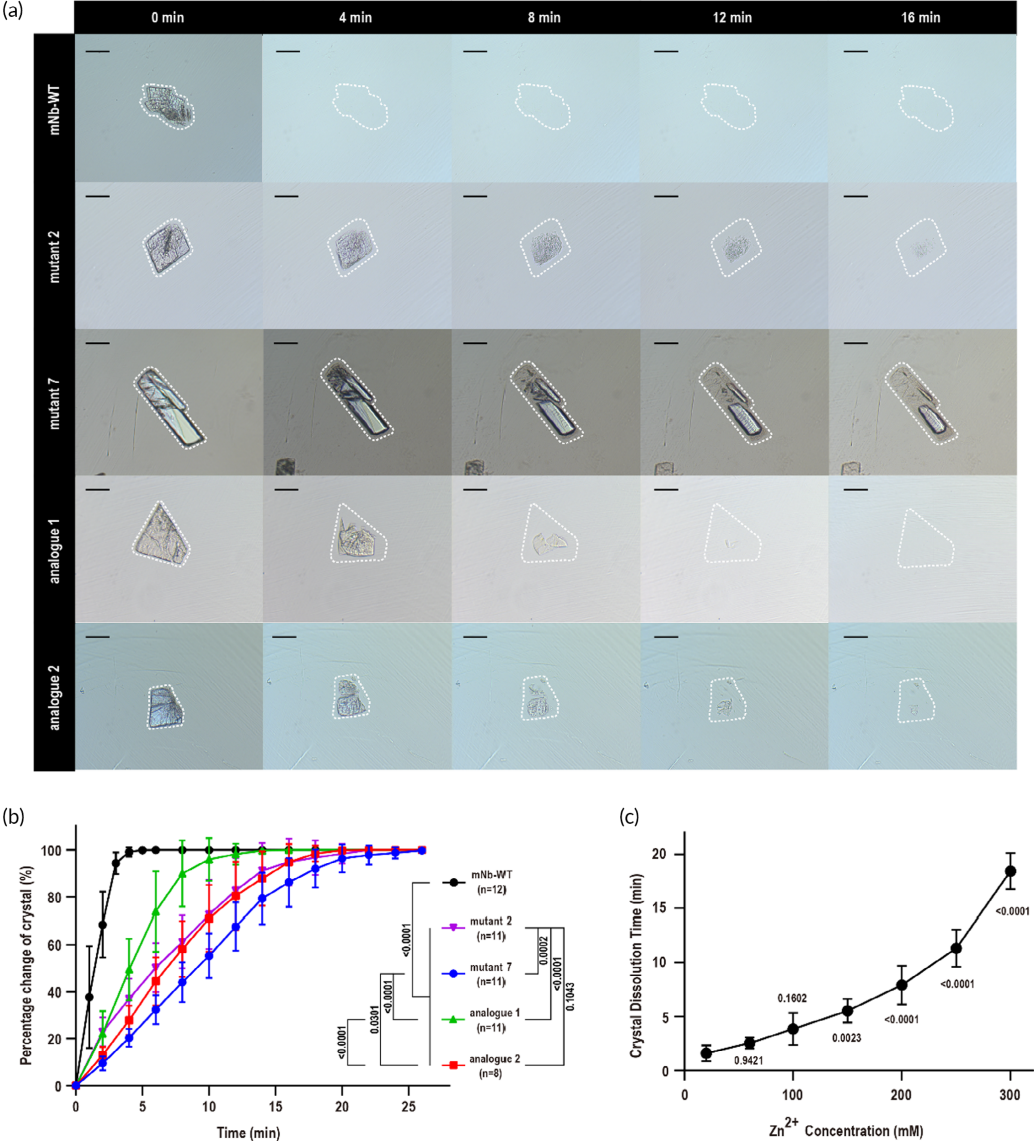
Protein release kinetics from different crystals. (a) Images of the dissolution process of different crystals. The scale bar represents 20 μm. (b) The percentage size change of the protein crystals relative to the initial size. The error bar is the standard deviation (SD). (c) The dissolution time of mutant 7 crystals in a linear gradient of zinc ions. The error bar is the SD. The *p* values are labeled in the figure, and the *n* numbers of the 20, 60, 100, 150, 200, 250, and 300 mM zinc ion groups were 5, 5, 6, 5, 7, 7, and 5, respectively.

To verify whether the crystal arrangement could affect the protein release kinetics from the crystals, we analyzed the trend of the two‐dimensional projected area of different crystals in the PBS (pH = 7.4) to reveal the release rate of each crystal. Limited by the tiny number of crystals of each protein, we photographed the dissolution process of each crystal in 10 μl PBS buffer at 2 min intervals. Then, we measured the two‐dimensional projected area of all of the tested crystals and recorded the percentage of area reduction at each time point. The images of the dissolution process of different crystals are shown in Figure 6a. Obviously, the size reduction rate of the protein crystals was significantly decreased to various degrees by different surface mutations, among which mutant 7 was the most effective (Figure 6b). Overall, the *P*2_1_2_1_2_1_ lattice was clearly superior to the *P*1 lattice. However, similar to the *P*1 lattice, the protein release of the mNb‐WT, analogue 1 and 2 crystals were significantly delayed as the solvent content of the crystal decreased. For the *P*2_1_2_1_2_1_ lattice, zinc coordination of the mutant 7 crystal further strengthened the molecular interaction, leading to a slower release rate than mutant 2 crystals. These results suggested that the protein release rate from crystals could be remarkably extended by a more compact arrangement of protein molecules or stronger internal interaction inside the crystal lattice. Constrained by the very limited amount of protein crystals, the protein release profile reported here was a semiquantitative evaluation of protein release kinetics from different crystals. A more precise and quantitative drug release method will be developed and implemented in the future, when sufficient amount of protein crystals are prepared in the later development stage. However, the rank order of drug release kinetics is expected to be the same as what was reported here in Figure 6b.

Considering the contribution of zinc ion in the lattice packing of mutant 7 protein, we wondered whether the release rate could be further modulated by the existence of zinc ion in the release medium. The dissolution of protein molecules from crystals should theoretically decelerate with zinc ions. Therefore, we detected the time for mutant 7 crystals to dissolve completely in release medium with a zinc ion concentration gradient. Indeed, increasing zinc ion concentration in the release medium, the dissolution time of mutant 7 crystals increased significantly (Figure 6c). By controlling the zinc ion concentration of the crystal suspension solvent, the release rate of mutant 7 crystals could be further modulated.

### 2.7 The crystallization propensity of nanobodies was affected by their *T_m_* and *T*_*agg*_

The thermodynamic properties of proteins depend on the protein primary sequence, and the protein crystalline propensity may be directly related to the thermodynamic properties of the protein. Therefore, we explored the possible connections between the crystallinity and the thermodynamic properties of different proteins. We recorded the crystal condition number of the mNb‐WT protein and mutants under the same crystal screening batch matrix. Analogue 1 and 2 proteins exhibited a higher number of crystallization conditions; while some mutants completely lost the crystallization capability, such as mutant 1, mutant 3, mutant 4, and mutant 5 (Table 2). The above crystal screening studies were duplicated and the results showed similar crystallization propensity of these nanobodies (data not shown).

**TABLE 2:** The crystal propensity of mNb‐WT and mutations

	S3	S6	S10	S20	S21	S22	S23	Total condition number
mNb‐WT	2	1	0	4	4	9	0	20
Mutant 1	0	0	0	0	0	0	0	0
Mutant 2	2	0	0	1	1	3	2	9
Mutant 3	0	0	0	0	0	0	0	0
Mutant 4	0	0	0	0	0	0	0	0
Mutant 5	0	0	0	0	0	0	0	0
Mutant 6	0	0	0	1	0	1	0	1
Mutant 7	0	0	0	0	0	1	0	1
Analogue 1	4	11	7	5	8	17	3	53
Analogue 2	3	4	1	5	5	10	1	28

*Note*: S3: Crystal Screen ½ kit, Hampton Research. S6: Index kit, Hampton Research. S10: JCSG plus kit, Molecular Dimensions. S20: JBScreen Basic HTS kit, Jena. S21: JBScreen Kinase HTS kit, Jena. S22: JBScreen Classic HTS I kit, Jena. S23: JBScreen Classic HTS II kit, Jena. The red numbers are crystallization condition numbers after combining the same condition components.

**FIGURE 7:**
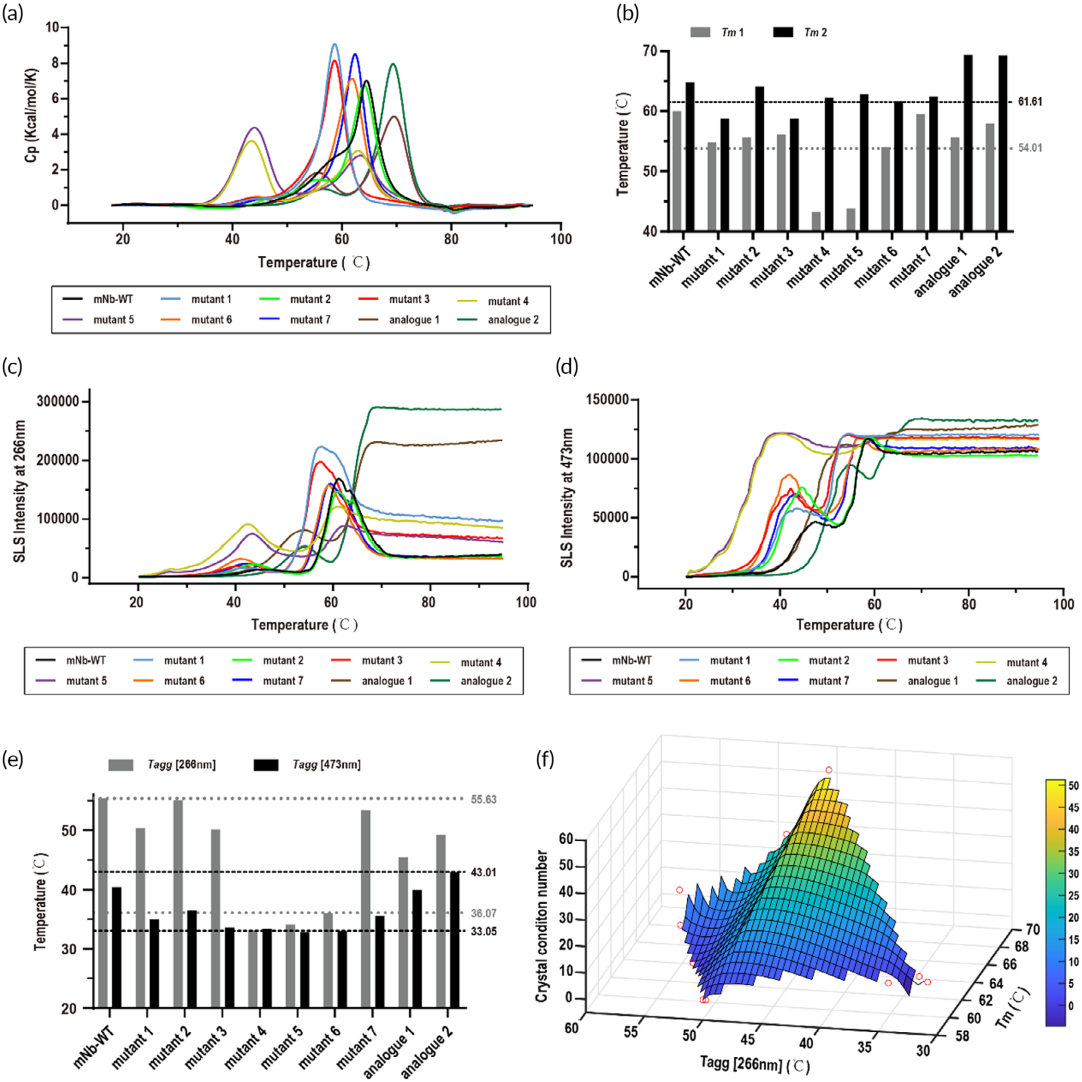
Relationship between thermodynamic properties and crystallization propensity. (a) The *T*_*m*_ curves of mNb‐WT and mutants 1–7 and analogues 1–2. (b) The *T*_*m*_ values of a. (c) The aggregation curves of tested proteins at 266 nm. (d) Aggregation curves of the tested proteins at 473 nm. (e) The *T*_*agg*_ values of (c) and (d). (f) The relationship between crystal numbers, *T*_*m*2_ and *T*_*agg*_ (266 nm). The tested sample was labeled as red circles.

We measured the unfolding temperature (*T*_*m*_) and aggregation temperature (*T*_*agg*_) of mNb‐WT and mutants/analogues to reveal the thermodynamic stability and aggregation propensity of each protein, respectively. The thermodynamic stability and aggregation tendency of proteins changed substantially by one or more mutations (Figure 7a–e). Every tested nanobody exhibited two unfolding events, and the protein structures were destroyed after the second unfolding event, making *T*_*m*2_ more responsive to protein thermodynamic stability (Figure 7b). As the beginning of crystallinity, aggregation plays a critical role in protein behavior. Small aggregates were observed at 266 nm by SLS, and large aggregates were detected by SLS at 473 nm. Small aggregates reflect the aggregation tendency of proteins, such as the protein oligomer assembly. By analyzing the *T*_*m*2_ and *T*_*agg*_ (266 nm) of the tested proteins, an interesting fact was demonstrated: proteins with higher *T*_*m*2_ and *T*_*agg*_ (266 nm) values around 45–50°C, appeared to crystallize more easily (Figure 7f). With a high *T*_*m*_ value (above 61.6°C), the proteins tended to aggregate into precipitants when *T*_*agg*_ (266 nm) was below 36.1°C; meanwhile, above 55.6°C, the aggregation tendency was too weak and proteins tended to remain in solution.

## 3 DISCUSSION

Although the concept of using protein crystals for long acting therapeutics was proposed earlier,^32^, ^33^ and pharmaceutical superiority was observed when high concentration of protein drugs were administrated as crystalline form,^20^, ^34^, ^35^ crystalline insulin remains the only marketed crystalline protein drug product.^36^ One of the major bottlenecks is that protein crystallization process is difficult to occur kinetically, and the protein crystallinity thus pharmaceutical properties were difficult to modulate.

With one or more mutations of surface residues, we successfully changed the thermodynamic properties of a model protein, leading to a shift in the crystallization propensity of pharmaceutical analogues with identical biological activities. With increasing *T*_*m*_ and a moderate *T*_*agg*_ value, the number of crystallization conditions of protein analogues gradually increased (Figure 7f), indicating the possibility that more crystallizable protein candidates with same efficacy could be acquired by rational mutations, and thermodynamic values such as *T*_*m*_ and *T*_*agg*_ could be used as readily assessable indicators for crystallization propensity. Certainly, this exciting prospect is hypothesized based on a limited sample size in this study. A concrete correlation between the crystallization properties of a protein and its primary sequence could only be established based on a much larger data library, which is currently undergoing with the assistance of machine learning.

Crystalline proteins could possess different pharmaceutical application perspectives, such as controlled release, improved stability, and so forth.^20^, ^34^, ^35^ In this study, pseudo “polymorphic” protein crystals could be obtained through rational surface mutations, subsequently, the protein release rate could be modulated by the different packing energy of the polymorphic crystal lattices. Besides, it was suggested that after protein molecules crystallize, some solvent‐exposed protein surfaces could be embedded into the crystal lattice to form water inaccessible protein contact interfaces, thus could further reduce the protein release rate. Furthermore, we showed that the dissolution rate of the zinc‐protein co‐crystals could be further modulated by the zinc concentration in the release medium (Figure 6). On the other hand, protein crystallization could also optimize the solvent‐exposed protein surface, reduce the molecular mobility, and prevent the unfolding of protein by fixing the side chains of surface residues,^18^ thus collectively enhance protein stability.

It is worth noting that, various approaches could be used to further improve the chance of crystallization and crystal quality of proteins, such as assisting crystallization through other protein stabilizers or ligands,^37^, ^38^ truncating the flexible region of target proteins,^39^ eliminating the post‐translational protein modification,^40^ introducing disulfide bonds to enhance protein symmetry into dimers;^41^, ^42^ or introducing potential metal coordination (nickel, copper, and zinc);^43^ and so forth. Through such approaches, the density and lattice energy of protein crystals could be further improved.

In summary, our findings demonstrated that the protein crystalline form can be modulated by rational surface mutation without impairing pharmaceutical efficacy. Besides, the expected superior stability,^18^ high protein concentration,^19^ and low viscosity,^20^ collectively make crystalline protein a very promising novel intravitreal drug delivery strategy for wet AMD therapy. In fact, this study also suggested that the pharmacological aspects (target binding affinity, biological activity, etc.) and the pharmaceutical aspect (crystallization propensity, formulation design, etc.) of a novel protein drug could be simultaneously considered once a protein design project is started. With such “product‐oriented drug discovery” mentality, an optimized final protein drug product could be obtained much more efficiently, compared with the traditional, step‐by‐step discovery and development process where various expected or unexpected challenges are to be resolved sequentially with little global and proactive designs.

## 4 MATERIALS AND METHODS

### 4.1 Materials

The chromatography columns were purchased from Cytiva. The recombinant VEGF‐A_165_ protein was purchased from SinoBiological Co. Ltd. (Cat: HPLC‐11066‐HNAH). All crystallization reagents were from Hampton Research Inc., Molecular Dimensions, Ltd., and Jena Bioscience, Inc. The HRP‐conjugated 6xHis tag antibody for ELISA was purchased from ProteinTech Co. Ltd. (Cat: HRP‐66005).

The tested protein sequences are displayed in Figure 2a. The model anti‐VEGF nanobody was termed “mNb‐WT,” which binds to endogenous VEGF protein.^44^ Mutants 1–7 were rationally designed based on the mNb‐WT nanobody. The two nanobody analogues were proprietary anti‐VEGF nanobodies; thus, the complete sequences were not disclosed.

### 4.2 Protein expression and purification

All of the tested proteins were expressed in *Escherichia coli* BL21 (DE3) competent cells to avoid the potential influence of posttranslational modification. The expression and purification procedure of all of the tested proteins were similar. The bacteria with constructed plasmids were cultured in lysogeny broth medium with 100 μg/ml ampicillin at 37°C until the OD_600_ reached 0.6, and isopropyl *β*‐d‐1‐thiogalactopyranoside (InalcoPharm., 1758‐1400) at a final concentration of 1 mM was employed to induce the expression of target nanobodies. At 6 h postincubation, the expressed target protein was enriched as inclusions in the cytoplasm of *E. coli*.

The bacteria were centrifuged at 4000 rpm/min for 10 min and resuspended in Buffer A (20 mM Tris–HCl, pH = 8.0, 150 mM NaCl). Then, the resuspended bacteria were lysed with a high‐pressure microjet homogenizer (ATS, AH‐TITAN PLUS) at 4°C. The protein inclusions were collected by centrifugation (12,000 rpm/min, 10 min). After two washes with Buffer B (20 mM Tris–HCl, pH = 8.0, 150 mM NaCl, 0.5% v/v Triton X‐100), the inclusions were redissolved completely in Buffer C (50 mM Tris–HCl, pH = 8.0, 250 mM NaCl, 6 M Gua‐HCl, 10% v/v glycerol) to 30 mg/ml at 4°C. With stirring, 7 ml inclusions in Buffer C were added dropwise into 500 ml Buffer D (100 mM Tris–HCl, pH = 8.0, 150 mM NaCl, 2 mM EDTA·2Na, 600 mM L‐Arg·HCl, 5 mM GSH, 0.5 mM GSSG) with a syringe needle at 4°C.

After 36 h, the refolded protein was concentrated and exchanged into Buffer E (20 mM Tris–HCl, pH = 6.8, 20 mM NaCl) by an Ultra Filtration Cup (Merck Millipore, UFSC40001) for source S column purification. Then, the protein peak, which was eluted with a linear gradient by Buffer F (20 mM Tris–HCl, pH = 6.8, 1 M NaCl), was collected and reloaded into a Superdex 75 10/300 column (GE Healthcare) preequilibrated with Buffer F (20 mM Tris–HCl, pH = 8.0, 150 mM NaCl). Once again, the protein peak fractions were collected and concentrated to 10 mg/ml for the following experiments. The concentration of protein was measured by a NanoDrop 2000 (Thermo Fisher Scientific) at 280 nm, and the purity of the peak fractions was detected by SDS‐PAGE.

### 4.3 Crystallization and data collection

The crystallization experiments were conducted as previously described.^45^ The proteins for the crystallization experiments were those without labels. The frozen protein was thawed on ice and centrifuged to remove the potential crystal nucleus and precipitants. The protein was mixed at a 1:1 ratio with commercial crystal condition kits with the sitting‐drop vapor diffusion method by the Protein Crystallization Screening System (TTP LabTech, mosquito). After optimization for a few days, the single crystal was successfully cultured at 21°C and flash‐frozen in liquid nitrogen. The diffraction data of different crystals were collected at the Shanghai Synchrotron Research Facility and autoprocessed with aquarium pipeline.^46^

### 4.4 MicroED sample preparation and data collection

The microED technique followed a previously published workflow.^47^, ^48^, ^49^, ^50^ Lacey carbon copper EM grids (200 meshes, EMCN) were used to prepare sample of mutant two crystals. A drop (3 μl) of the suspended crystals was loaded onto a glow‐discharged grid then placed horizontally for 1 min. Vitrobot Mark IV (Thermo Fisher Scientific) was used to perform plunge‐freezing. The grid was loaded to Vitrobot, blotted for 30 s at 50% humidity and room temperature, then plunged to liquid ethane cooled in liquid nitrogen.

Briefly, the frozen grids were loaded to a dual‐beam FIB‐SEM system (Helios NanoLab DualBeam G3 UC, Thermo Fisher Scientific) equipped with a cryo‐stage (PP3010T, Quorum). The grids were sputter‐coated with a layer of conductive platinum then deposited with a layer of organometallic platinum using the gas injection system. Crystals were milled to 200 nm in multiple steps using decreasing ion currents from 0.79 nA to 40 pA.

The FIB‐milled grids were mounted to a Gatan 626 cryo‐holder at cryogenic temperature and transferred to 200 kV Tecnai F20 TEM (Thermo Fisher Scientific) equipped with a Gatan US4000 CCD camera. The eTasED software package was used to perform semi‐automated MicroED data collection. Each image was exposed 5.72 s at an electron dose rate of 0.005 e^−^/(Å^2^s) at the same time of 1.0° rotation. A selected‐area aperture (diameter of 200 μm, equivalent to ~5 μm on the object plane) was used in data collection.

All structures were determined by the molecular replacement method.^51^ Model building and structural refinement were performed using COOT and PHENIX software.^52^, ^53^

### 4.5 ELISA

The proteins for biological activity were labeled with a 6xHis tag. The VEGF‐A_165_ protein was coated on high adsorption plates and incubated overnight at 4°C. After blocking with 5% w/v skim milk for 2 h, the plates were incubated with a linear gradient of nanobodies for 1 h at 37°C. After washing the tested wells with 0.5% v/v Tween 20‐PBS buffer, the HRP‐conjugated 6xHis tag antibody was employed for nanobody binding. The fluorescent signal was displayed by TMB solution and detected with a multifunctional readout (Thermo Fisher Scientific, VARIOSKAN FLASH) at 450 nm.

### 4.6 Circular dichroism spectrum analysis

All of the tested nanobodies for the circular dichroism (CD) spectrum (Applied Photophysics, Chirascan plus) were expressed without purifying tags and diluted to 0.25 mg/ml in the same buffer (20 mM Tris–HCl, pH = 8.0, 150 mM NaCl). The CD spectrum signal was recorded in the range of 200–260 nm for all proteins at 25°C. The step was 1 nm, and the scanning time per point was 0.5 s. The content of the secondary structure of all the proteins was calculated by CDNN software.

### 4.7 Protein release assay

Protein release from the crystals was conducted in 10 μl release medium at 25°C. The tested crystals were extracted by a crystal loop into wells of a 24‐well sitting‐drop vapor diffusion plate filled with 10 μl 1x PBS buffer, surrounded by 1 ml release medium, and covered by tape to avoid excessive evaporation. Subsequently, the crystals were imaged by a microscope (Carl Zeiss) in a bright field at 2 min intervals. The crystal images of all of the time points were loaded into ImageJ software to measure the 2D projected area. Then, we calculated the remaining percentage of tested crystal at each time point compared to the initial 2D area. The statistics were calculated with one‐way ANOVA using GraphPad Prism software.

The protein release assay of mutant 7 crystals with a zinc ion gradient was performed using a similar method. Due to the specificity of zinc ions, we changed the release medium from 1x PBS to 100 mM MES buffer (pH = 6.5) with an increasing gradient of zinc acetate (Sigma, 379786‐5 g). After transplanting mutant 7 crystals into the release medium well, the time required to completely dissolve the crystals was recorded. The statistics of dissolution time were calculated by one‐way ANOVA using GraphPad Prism software.

### 4.8 Determination of the unfolding temperature of proteins

The proteins used for *T*_*m*_ determination were expressed without purifying tags. After centrifugation at 12,000 rpm/min for 10 min, the proteins were diluted to 1.25 mg/ml with the same buffer (20 mM Tris–HCl, pH = 8.0, 150 mM NaCl), and the protein concentration was precisely confirmed with a NanoDrop 2000 (Thermo Fisher Scientific) at 280 nm. A microcalorimetry scanning calorimeter (Malvern Panalytical Ltd, MicroCal PEAQ‐DSC) was employed to scan the caloric change between the protein sample and buffer reference during programmed heating in the range of 15–100°C at 90°C/h. The *T*_*m*_ point was determined by MicroCal PEAQ‐DSC analysis software.

### 4.9 Determination of aggregation temperature of proteins

The proteins were used for *T*_*agg*_ point determination without a 6xHis tag. The proteins were centrifuged at 12,000 rpm/min for 10 min to remove the possible precipitants. The SLS signal of the tested protein was detected at 266 and 473 nm for the small assembly and large aggregation, respectively, during the programmed heating process at 1.5°C/min in the range of 15–95°C with a protein stability analyzer (Unchain Lab Ltd, UNcle). The onset of SLS curves was determined as the *T*_*agg*_ point at 266 and 473 nm. The *T*_*agg*_ point was calculated using UNcle analyzer software.

### 4.10 Statistical analysis

All the experiments were conducted at least three times except the data collection and structure refinement of nanobody crystals. The images of crystal dissolution were the representative of each tested crystal group. The statistical significance was determined by GraphPad 8.0 software. The *n* number and *p* value of each tested group were labeled in the figures, and the error bars were represented by SD.

No unexpected or unusually high safety hazards were encountered throughout this study.

## AUTHOR CONTRIBUTIONS

Shuqian Zhu: Conceptualization (lead); data curation (lead); investigation (lead); visualization (lead); writing‐original draft (lead); writing‐review and editing (lead). Shilong Fan: Data curation (equal); visualization (equal). Tianxin Tang: Data curation (supporting). Jinliang Huang: Data curation (supporting). Heng Zhou: Data curation (supporting). Chengnan Huang: Data curation (supporting). Youxin Chen: Conceptualization (equal); supervision (supporting). Feng Qian: Conceptualization (lead); investigation (equal); supervision (lead); writing‐review and editing (equal).

## CONFLICT OF INTEREST

The authors declare that no conflict of interests.

### PEER REVIEW

The peer review history for this article is available at https://www.webofscience.com/api/gateway/wos/peer-review/10.1002/btm2.10523.

## Supporting information


**TABLE S1.** The data collection and refinement statistics of “polymorph” crystals. (PDF)

## Data Availability

All data except for the sequence of analogues 1 and 2 are available in the main text or the supplementary materials. Sequence of analogues 1 and 2 will become available once an confidential agreement was signed.
