# Supplementary material for: Polymorphic nanobody crystals as long‐acting intravitreal therapy for wet age‐related macular degeneration
Source: Bioeng Transl Med. 2023 May 4;8(6):e10523. doi: 10.1002/btm2.10523 (PMC10658565; doi:10.1002/btm2.10523)
Supplement: Supplementary file 1 — TABLE S1. The data collection and refinement statistics of “polymorph” crystals. (PDF) [file BTM2-8-e10523-s001.docx]

**Supplementary materials**

**Table S1. The data collection and refinement statistics of “polymorph” crystals**

|  | **mNb-WT** | **mutant 2** | **mutant 7** | **analogue 1** | **analogue 2** |
| --- | --- | --- | --- | --- | --- |
| **PDB code** | **8IIU** | **8IJZ** | **8IJS** | **/** | **/** |
| **Dissection Method** | **X-Ray** | **micro-ED** | **X-Ray** | **X-Ray** | **X-Ray** |
| **Data Collection** |  |  |  |  |  |
| Source | BL18U1 | FEI Tecnai F20 | BL18U1 | BL10U2 | BL10U2 |
| Wavelength (Å) | 0.9897 | 0.02507 | 0.9897 | 0.9796 | 0.9796 |
| Space Group | *P*1 | *P*2_1_2_1_2_1_ | *P*2_1_2_1_2_1_ | *P*1 | *P*1 |
| **Cell Dimensions** |  |  |  |  |  |
| a, b, c (Å) | 31.468 39.369 39.952 | 31.22  41.09  74.05 | 24.915 53.205 65.376 | 30.326 38.419 38.781 | 30.381 38.201 38.773 |
| α, β, γ (˚) | 102.14 90.231 90.948 | 90  90  90 | 90  90  90 | 101.846 90.036 92.225 | 102.137  90.1  92.211 |
| Resolution (Å) | 24.59-1.27 (1.315-1.27) | 16.72-2.1 (2.175-2.1) | 20.17-1.752 (1.814-1.752) | 24.32-1.414 (1.465-1.414) | 19.21-1.19 (1.233-1.19) |
| Unique Reflections | 46508 (4516) | 4937 (463) | 9121 (781) | 31139 (3033) | 50684 (4867) |
| Completeness (%, highest shell) | 94.01 (91.16) | 83.1 (80.8) | 98.51 (86.01) | 95.17 (93.12) | 92.68 (87.93) |
| Redundancy | 3.4 (3.3) | 9.57 (9.41) | 11.8 (7.9) | 2.4 (2.5) | 3.3 (2.6) |
| I/σI (highest shell) | 16 (1.53) | 7.72 (2.28) | 41.85 (1.27) | 6.8 (4.7) | 9.6 (2.3) |
| CC1/2 | 97.4 (74.3) | 99.5 (35.0) | 99.7 (65.4) | 90.3 (52.8) | 99.6 (84.2) |
| Wilson B-factor | 11.53 | 42.2 | 31.72 | 10.87 | 11.62 |
| **Refinement** |  |  |  |  |  |
| Resolution (Å) | 1.27 | 2.1 | 1.75 | 1.414 | 1.19 |
| Number of Reflections | 46505 (4515) | 4937 (463) | 9120 (781) | 31135 (3033) | 50671 (4865) |
| Reflections Used for  R-free | 1994 (202) | 247 (23) | 913 (78) | 2014 (195) | 1985 (182) |
| R-work | 0.1669 (0.2111) | 0.2329 (0.2705) | 0.2005 (0.2925) | 0.1899 (0.2168) | 0.1873 (0.2869) |
| R-free | 0.1869 (0.2714) | 0.3458 (0.2981) | 0.2214 (0.3728) | 0.2077 (0.2401) | 0.2215 (0.3404) |
| **Number of Atoms** |  |  |  |  |  |
| Macromolecules | 1925 | 934 | 900 | 1902 | 1957 |
| Ligands | 10 | 0 | 2 | 10 | 10 |
| Water | 313 | 0 | 41 | 262 | 257 |
| **RMSDs** |  |  |  |  |  |
| Bond Lengths (Å) | 0.005 | 0.01 | 0.006 | 0.017 | 0.021 |
| Bond Angles (°) | 0.91 | 1.22 | 0.97 | 1.74 | 1.79 |
| **Reamachandran Plots** |  |  |  |  |  |
| Favored (%) | 97.11 | 97.52 | 97.30% | 99.17 | 98.77 |
| Allowed (%) | 2.89 | 2.48 | 2.70% | 0.83 | 1.23 |
| Outliers (%) | 0 | 0 | 0 | 0 | 0 |
| Clashscore | 5.51 | 7.09 | 6.79 | 9.57 | 19.37 |
| **B-factors** |  |  |  |  |  |
| Average B-factor | 17.35 | 44.96 | 44.44 | 15.93 | 16.41 |
| Macromolecules | 15.31 | 44.96 | 44.24 | 14.52 | 15.18 |
| Ligands | 19.86 | 0 | 73.09 | 18.7 | 21.49 |
| Solvent | 29.76 | 0 | 47.62 | 26.1 | 25.54 |
